# Medical Miscellany

**Published:** 1853-12

**Authors:** 


MEDICAL MISCELLANY.

It should be known that if persons in a house on fire have the presence of mind to apply a damp cloth or handkerchief to their mouths and nostrils, they can effect a passage through the densest smoke—but the surest way would be to envelope the head and face completely in a damp cloth.——Dr. Stephenson, of Indiana, gave a deaf dog two grains of strichnia, intending to kill him: the next day, so far from being dead, he had entirely recovered from his deafness, and has ever since heard as well as other dogs.——It is stated that a man was lately at Cincinnati who eats no cooked food, of any kind, and alleges that he has lived on raw meat, potatoes and vegetables, for several years.——A spotted child was recently born in N. H. One half of the head, including half of the forehead, is black, while the other half is white: the face, below the eye-brows, assumes an ash-yellow. The shoulders are spotted; the rest of the body is white.——It is stated in the life of Sir Astley Cooper, that his professional income was at one time, one hundred thousand dollars: more than was probably ever acquired, in the same time, by any member of the profession. At that rate, his income amounted to nearly $300 a day.——The New York Hospital has 360 beds, of which 250 are devoted to surgical cases, most of them of recent occurrence. Bellevue Hospital has 630 beds, about 450 of which are now occupied.——Recently published statistics show that 32,000 children are born in Paris annually, one third of which are illegitimate.——Mrs. Webster, widow of the unfortunate Prof. W., died recently at Cambridge.——Mrs. Thos. Seely, aged 90, residing at Neversink, it is said, became the mother of a living child on the first week of August, and it is doing well.——A committee of four physicians is completing the second part of the second volume of Dr. Pereira's Materia Medica: the greater part was finished and in press at the time of Dr. P.'s death.——Dr. Aran states that 150 drops of chlorolorm may be safely administered in cases of colic, at proper intervals, with the most favorable results.——The American Pharmaceutical Association held its annual meeting at Boston, in August.——Dr. Tinsley, of Cuba, claims to have discovered that "vaccine virus, after passing through the system of a negro, is valueless to the white race.."——Dr. Toynbee, of England, has lately received a prize for the application of art to medical purposes, in the invention of an artificial membrana tympani, used in cases of deafness from want of that organ.——Dr. Geo. C. Shattuck, of Boston, lately gave $14,000 to Harvard University, to found a Professorship of morbid Anatomy, in the Medical College.

